# The Style of Coping with Stress, the Health-Related Locus of Control, and the Level of Mindfulness of Patients with Chronic Somatic Diseases in Comparison to Healthy People

**DOI:** 10.3390/healthcare10091752

**Published:** 2022-09-12

**Authors:** Magdalena Gruszczyńska, Monika Bąk-Sosnowska, Anna Daniel-Sielańczyk, Julia Wyszomirska, Adriana Modrzejewska

**Affiliations:** Department of Psychology, Chair of Social Science and Humanities, School of Health Sciences in Katowice, Medical University of Silesia in Katowice, 40-752 Katowice, Poland

**Keywords:** health locus of control, mindfulness, chronic disease, psychological factors, stress coping, healthy people

## Abstract

Background: Psychological factors may be involved in both the development and treatment of somatic diseases. Our study aimed to evaluate the style of coping with stress, health-related locus of control, and level of mindfulness of adult patients with the most common chronic somatic diseases compared with healthy subjects. Methods: 502 chronically ill people were examined (M = 49.27; SD = 14.25), including 288 women and 214 men. The control group consisted of 117 healthy people (M = 45.66; SD = 17.86). The diagnostic survey involved using the Coping Inventory for Stressful Situations (CISS), Multidimensional Health Locus of Control Scale (MHLC), and Mindful Attention Awareness Scale (MAAS). Results: Differences between the clinical and control groups were demonstrated for MHLC: Internal (*p* < 0.001), Powerful Others (*p* < 0.05), and Chance (*p* < 0.001); CISS: Task (*p* < 0.001) and Avoidance (*p* < 0.05); and MAAS (*p* < 0.01). Conclusion: People with chronic somatic diseases, compared to healthy people, have a stronger external and weaker internal health-related locus of control, lower level of task and avoidance style for coping with stress, and lower level of mindfulness.

## 1. Introduction

Any chronic disease is characterized by a long duration and slow progression of lesions [1]. Due to the growing number of people struggling with various types of chronic diseases, the burden on the health care system and the home budgets of individuals and families has increased around the world [2,3]. For years, scientists have been developing, modifying, and implementing various types of treatments and procedures to prevent the increase in the incidence of chronic diseases and, in people who are already ill, further progression of the disease. This often involves implementing interventions aimed at permanently changing lifestyles [4,5].

Over the last decades, a biomedical model has been used, which focuses on the treatment of a specific disease entity without any accompanying diseases and psychological aspects of the patient’s functioning. This model ignores an important therapeutic factor: the patient’s motivation for treatment, adaptation possibilities, attitude towards the disease, and coping ability with a stressful situation [6]. It should also be remembered that a patient may suffer from more than one disease simultaneously, which significantly reduces their coping abilities, and that state affects the treatment process [7,8]. Based on the above observations, a broader, holistic view of the patient’s situation became important, which initiated the development of the biopsychosocial model. It also required taking into account a larger number of people in the therapeutic process (doctors of various specialties, support staff, and psychologists) and, above all, the patient. Thanks to that, the patient became the subject and not the object of treatment [9].

The role of psychological factors in disease development and treatment is appreciated, although the area is not fully explored yet. Taking into account the complexity of the human psyche, the cultural and social context, age, and gender, many variables may differentiate the way patients function. The disease is an evident stressor, which in the case of chronic disease is also chronic due to longevity, and it often involves introducing necessary though burdensome lifestyle changes [10,11]. The disease affects many aspects of life. Depending on its advancement and duration, it will be life-limiting to a greater or lesser extent. Chronic disease is characterized by, among other things, the unpredictability of symptoms, which significantly increases the level of stress [12]. How the patient copes with the disease and its symptoms and limitations and which coping strategies are triggered in this process will affect, inter alia, the quality of patient life [10,12,13]. The style of coping with stress is understood as a combination of cognitive and behavioral efforts aimed at controlling, reducing, or eliminating stress [14]. The following styles of coping are distinguished: task-oriented style, which consists in focusing on the problem and actively seeking information and solutions; emotion-oriented style, which is associated with lowering stress through emotional reactions (e.g., anger or blame) or rumination; and avoidance-oriented style, which refers to distraction and withdrawal to avoid stress and may take the form of substitute activities or excessive social involvement [14].

Patients struggling with a chronic disease, which, as mentioned above, is unpredictable, often feel that they have lost control over their health and life. The reason is the length and, often, the nuisance of symptoms and changes in everyday life resulting from the disease. Therefore, an extremely important aspect of treatment is to restore patients’ sense of control, so they become participants in the process, not only the passive recipient. In the process of restoring control, it is important to identify the health-related locus of control (HLC), which refers to beliefs about the sources of influence on one’s health. Patients believe that their health depends only on them (internal HLC), on external factors such as authorities and doctors (powerful others HLC), or chance (HLC) [15]. Location of health control is one of the indicators used to test patients’ health beliefs and is associated with self-care and leading a healthy lifestyle [16].

When looking for factors and methods that can help in the treatment of chronic diseases, attention has been paid to the mindfulness-based approach. In recent years, this technique has been introduced to reduce stress in people suffering from somatic and mental illnesses [17,18]. Mindfulness is a kind of deliberate, non-judgmental attention to the present [19]. Research indicates the significant effect of mindfulness on stress reduction, adaptation to disease, and reduction of pain sensations in various types of chronic diseases [20,21,22,23].

Previous studies insufficiently analyzed the level of psychological variables mentioned above in chronically ill people, comparing them with the level of these variables in healthy people. On the other hand, many studies proved that each of these variables is related to health. A holistic view of patients, their physical illness, and psychosocial functioning allow healthcare professionals to include these individual variables in creating patient-centered care and formulate therapeutic recommendations according to the psychological characteristics of the patient.

Our study aimed to evaluate the style of coping with stress, the health-related locus of control, and the level of mindfulness of adult patients with the most common chronic somatic diseases compared with healthy subjects. Based on the scientific literature and our clinical practice, we formulated the main research hypothesis: there are differences in the level of selected psychological variables between chronically ill and healthy people. We also adopted the following detailed hypotheses:

1. Patients with chronic diseases have a significantly higher external (Powerful Others) locus of health control than healthy people

2. Patients with chronic disease have a significantly higher level of emotion and avoidance and a lower level of the task-oriented style of coping with stress than healthy individuals

3. Patients with chronic disease have a significantly lower level of mindfulness than healthy subjects

## 2. Materials and Methods

### 2.1. Participants

Ultimately, 502 chronically ill people (clinical group), including 288 women and 214 men, participated in the study. The mean age of the respondents was 49.27 ± 14.25 years. The inclusion criteria for the study were 18 years old or over, at least six months from the diagnosis of chronic disease, and given recommendations to use pharmacotherapy for chronic disease. Familiarization with the purpose of the study and then completing and voluntarily returning an anonymous questionnaire was tantamount to giving informed consent to participate. The exclusion criteria were diagnosed dementia, current acute depressive or psychotic episode, and psychophysical state enabling participation in the study.

The control group consisted of 117 (M = 45.66; SD = 17.86) people, who did not differ significantly in sex (*p* = 0.628) or age (*p* = 0.082) from the clinical group. Inclusion criteria for this group were 18 years old or over and informed consent to participate in the study. The primary exclusion criterion was chronic disease diagnosis, while the remaining criteria were identical to those in the clinical group.

### 2.2. Method

In a cross-sectional study, three standardized tools were used to estimate psychological variables. These tools were selected due to their appropriate psychometric characteristics and adaptation to Polish conditions. Sociodemographic and health-related data were collected using a self-questionnaire, which took into account such variables as gender, age, education, place of residence, partner status, children, professional status, attitude to faith, chronic diseases, and pharmacotherapy recommendation.

The style of coping with stress was analyzed with the Coping Inventory for Stressful Situations (CISS) [24]. It contains 48 simple statements describing three groups of behavior focused on tasks, emotions, and avoidance, presented by people when they find themselves in difficult and stressful situations. Each is described by 16 items. The respondent determines the frequency of a given reaction by marking the appropriate number on a five-point scale (where 1 means never, 2 very rarely, 3 sometimes, 4 often, and 5 very often). The styles of coping with stress in the questionnaire are presented in the form of three subscales: task-oriented style (TOS), emotion-oriented style (EOS), and avoidance-oriented style (AOS). Additionally, two forms of avoidance were distinguished in the last subscale: Distraction Style (DS) and Social Diversion Style (SDS). Cronbach’s alpha reliability coefficient was 0.83 for TOS and EOS, 0.69 for AOS, 0.65 for DS, and 0.57 for SDS.

In order to determine the location of health control, the Multidimensional Health Locus of Control Scale (MHLC) was used, which was adapted for Polish conditions by Z. Juczyński [25]. The MHLC questionnaire contains 18 statements concerning generalized expectations related to the three dimensions of the location of health co: Internal (I)—control over own health depends on me; Powerful Others (PO)—own health is the result of the influence of others, mainly medical personnel; Chance (Ch)—health is determined by chance or other external factors. By marking a scale from 1, I completely disagree, to 6, I agree, the respondent defines to what extent he or she agrees with the given statements. The range of points that can be obtained in each of the three dimensions is from 6 to 36 points. High results in a given subscale indicate a strong belief in the impact of a given dimension on health. The reliability of the tool in the study is 0.73 for I, 0.67 for PO, and 0.62 for Ch.

The level of mindfulness has been determined using the Polish adaptation of the Mindful Attention Awareness Scale (MAAS) [26]. The MAAS questionnaire measures a person’s tendency to be alert and aware of a given moment in everyday activities. It consists of 15 questions. The greater the sum of points, the greater the degree of mindfulness an individual has. The respondents respond to 15 statements by marking the answers on a six-point Likert scale from “almost always”, through “very often”, “relatively often”, “relatively rarely”, “very rarely”, to “almost never”. The test result is in the range of 15–90 points. The Cronbach’s alpha of the scale was within the range of 0.89.

### 2.3. Study Organization

The study was conducted in five randomly selected primary health care units in Silesia, Poland. Participation in the study was offered to each adult patient who came for a medical visit during the three months of the study. The participants were informed about the purpose of the study, its anonymity, and voluntariness. People who agreed to participate in the study completed the questionnaires independently while waiting for a medical appointment or after one. The response time was not limited, and participants did not receive remuneration for participation. Returning the completed questionnaires to a specially prepared, secured box standing at the reception desk was tantamount to giving informed consent to participate in the study.

The institutions participating in the study received a total of 1000 questionnaires, and 843 questionnaires were returned to the researchers. After taking into account the exclusion criteria and the rejection of incomplete questionnaires, 651 remained, of which 534 belonged to the clinical group and the remaining 117 belonged to the control group.

### 2.4. Statistical Analysis

IBM SPSS Statistics (Version 27) was used to analyze the results. The Kolmogorov-Smirnov test was used to assess the normality of the distribution. The analysis of variance allowed for the assumption of the homogeneity of the variance. Due to the non-normal distribution of the examined variables, the non-parametric Mann-Whitney U test was used to calculate intergroup comparisons. The level of significance was set at α = 0.05.

## 3. Results

Most of the respondents were women (57.4%), city dwellers (73.9%), people with secondary and higher education (65.8), professionally active (52.6%), in a relationship (78.9%), having children (79.8%), and believers (90.2%). Details are presented in Table 1.

People from the clinical group identified the following dominant chronic disease: neoplastic disease (27.5%), rheumatoid arthritis (18.1%), obesity (12.5%), multiple sclerosis (12%), diseases of the musculoskeletal system (11.1%), cardiovascular diseases (9.4%), and diabetes (9.4%). Table 2 presents the descriptive statistics of the analyzed psychological variables.

The analysis of differences between the clinical group and the control group showed that the clinical group obtained lower scores on the following variables: internal locus of control (U = 23.791; *p* < 0.001), task-oriented (U = 22.421; *p* < 0.001), and avoidance-oriented (U = 25.521; *p* < 0.05) style of coping with stress, and level of mindfulness (U = 24.603; *p* < 0.01). The same group scored higher in Powerful Others (U = 26.086; *p* < 0.05) and Chance locus of control (U = 22.837; *p* < 0.001). Details of the analysis are presented in Table 3.

## 4. Discussion

Chronic disease, due to its long duration and, often, the nuisance of symptoms, is a stress factor that can significantly affect the quality of human life [11,12]. Identifying factors that can influence the effectiveness of a treatment may contribute to more adequate approaches to patients, taking into account their needs and possibilities.

The aim of our study was to evaluate the style of coping with stress, t-related locus of control, and the level of mindfulness of patients with chronic somatic diseases compared with healthy subjects. In the clinical group, we recorded a significantly lower level of internal health-related locus of control. Similarly, Boddu et al., using the same tool, compared 100 people with epilepsy with 70 healthy people and concluded that sick people had a lower level of internal health control [27]. Gibek’s research on 204 people suffering from chronic diseases, including oncological and cardiological diseases, showed that the group of patients was characterized by a lower level of internal health-related locus of control compared to healthy people [28].

People who are convinced that their health depends primarily on themselves more often engage in preventive and pro-health behaviors, i.e., beneficial in terms of lifestyle, nutrition, or physical activity [29]. Therefore, their risk of developing chronic disease is potentially reduced. It can, therefore, be assumed that people with a higher internal health-related locus of control found themselves in the control group because of greater responsibility for their health. Studies by other authors confirm that people with a higher level of internal locus of control may be in better health than people who, in assessing this factor, achieve a lower level [30]. Moreover, studies indicate that in healthy people who do not undertake treatment, the location of health control may be more internal, precisely due to the lack of experience in treatment [31].

In the case of chronically ill people, internal health-related locus of control is equated with increased motivation for treatment and changing their lifestyle to a more healthy one. Research indicates that the effectiveness of medical procedures and compliance with medical recommendations are also associated with patients taking responsibility for their health [16,32]. Moreover, Stainsby et al. showed that patients with a higher level of internal control are characterized by greater independence in dealing with the disease and less often need and rely on the help of others [33].

In our study, we showed that the chronically ill respondents had a higher level of external health-related locus of control compared to healthy people—both in terms of the influence of others and chance. In clinical practice, the attitude of patients, often resulting from their external locus of control, who experience the disease as a punishment, injustice, or malicious fate can be noticed. They avoid confrontation with the diagnosis, they do not accept the disease, and they do not undertake treatment efforts. At the same time, externally oriented patients shift the entire responsibility for treatment onto doctors, which, in turn, weakens their sense of influence and worsens the effects of therapeutic measures [34]. Although the location of health control is considered a weakly modifiable variable, it may change over time. For example, as a result of a chronic disease causing constant reliance on physicians and their prescriptions, the initial internal location of a patient’s control may shift to a more external one. It should be noted, however, that changes of this type take place over many years [31].

Patients with an external health-related locus of control will require motivational reinforcements from doctors and medical staff [35]. They are more inclined to follow the authority’s recommendations, which is why how doctors provide recommendations and how professional and consistent they are when communicating are so important [36]. It may also be important what pro-health attitude the doctor presents. Patients with an external health-related locus of control not only hand the responsibility for the success of treatment to doctors but also, when the results are not satisfactory, blame them [37]. Therefore, it seems reasonable to share the responsibility for the treatment process between the doctor and the patient, according to the competencies of each person. It may also be beneficial to use the support of a psychologist to strengthen the patient’s internal motivation for treatment [31].

In addition to the health-related locus of control, the style of coping with stress is an extremely important factor in the treatment process [38]. Research indicates a relationship between the location of control and the styles of coping with stress [39]. It turns out that patients who use less adaptive forms of coping with stress have a lower sense of agency and sense of internal control [40]. Similarly, in our study, the group of chronically ill people showed a lower level of task-oriented coping with stress compared to healthy people. The task-oriented style is related to activities aimed at effectively confronting the stressful situation, which in the discussed case is the disease and the treatment process [41]. Our results seem consistent with the fact that the clinical group presented at the same time a low level of internal health-related locus of control. It can be assumed that since people from the clinical group do not have an internal conviction about the impact on their health, they instead believe that it depends on others or on a case-by-case basis; therefore, they will be less task-oriented in dealing with this situation.

In our study, chronically ill people were also characterized by a lower avoidance-oriented style of coping with stress compared to healthy people. This result is inconsistent with the expected result, but other authors observed a similar situation. For example, Barańska et al. showed that patients suffering from osteoporosis presented the avoidant style the least frequently among all the styles of coping with stress. The higher the assessment of the individual perception of one’s health, the more often the respondents used active coping strategies [42]. Each disease is characterized by a variety and variable severity of symptoms, which may modify the experience as stressful. The level of a patient’s adaptation to chronic disease will significantly modify the level of stress. [43]. Research shows differences in the style of coping with stress in people suffering from various chronic diseases. Zarbo et al., by reviewing nine articles concerning, inter alia, styles of coping with stress in women with endometriosis, concluded that it is impossible to identify one dominant style of coping with stress [44]. On the other hand, Brands et al., when examining 310 people with chronic multiple sclerosis, identified the dominant task-oriented style for this group [45]. Perhaps if we examined the differences between our participants with certain chronic diseases, we would find different styles of coping with stress. This is a challenge for another study.

The group of chronically ill patients we studied also showed a lower level of mindfulness compared to the control group. Taking into account the previous variables, the result seems reasonable. Since sick people do not focus their attention on the “here and now”, they do not focus on the disease or the healing process, and their level of awareness is low. This is alarming from the point of view of studies showing the impact of mindfulness on stress reduction, disease adaptation, and the reduction of pain sensations and depression symptoms in various chronic diseases [20,23,46,47]. On the other hand, it is worth noting that mindfulness is one of those constructs that can be significantly modified through exercise and work on oneself, which is why it would be so important to strengthen the level of mindfulness in chronically ill patients. Boyle et al. examined 71 women with breast cancer [46]. The authors focused on two distinct strategies to regulate emotions: rumination and kindness. They studied the effects of mindfulness on these two variables. They showed that the six-week mindfulness training contributed to a decrease in rumination as well as an increase in kindness and mindfulness and, additionally, mediated in reducing stress and reducing depressive symptoms, which is extremely important in the face of chronic diseases.

In summary, it is important that medical personnel are trained in effective management of patients’ emotions, coping with stress strategies, and mindfulness, to be able to effectively help them overcome the anxiety associated with being ill and improve their quality of life. Since both the health-related locus of control and the style of coping with stress should be treated as a permanent predisposition, it may be important to adapt communication strategies individually to the patient. It may also be important, especially in the case of therapeutic difficulties, to cooperate with a team of specialists, doctors, nurses, and psychologists to identify the psychological functioning of the patient and select appropriate interventions.

At the same time, we are aware of the limitations of our research. They concern, inter alia, the lack of answers to questions about the time elapsed since the diagnosis, the stage of the disease, the severity of the symptoms, the prescribed pharmacotherapy, or patient adherence. As a result, we do not have data on the medical history of patients. All collected data were based on participants’ declarations, not medical records. We asked the patients to indicate the dominant disease subjectively. This was due to the anonymity of the study and the belief that in the study of psychological factors, the subjective assessment of the patient’s health and disease is more important than the objective state. Moreover, we did not analyze the patients’ responses depending on the type of their disease, which, as the previously cited studies show, may differentiate the image of psychological variables. We also did not study the relationship between the studied variables, which could interestingly broaden the perspective but was not consistent with the main goal of the study.

## 5. Conclusions

Chronically ill people, compared to healthy people, are characterized by a stronger external and weaker internal health-related locus of control. Moreover, they have a lower level of task-oriented and avoidance-oriented styles of coping with stress and a lower level of mindfulness.

## Figures and Tables

**Table 1:** Sociodemographic characteristics of the respondents.

		Clinical Group		Control Group	
Variable		*N*	%	*N*	%
Gender	Female	288	57.4	70	59.8
	Male	214	42.6	47	40.2
Dwelling-place	Rural	131	26.1	29	24.8
	Urban	371	73.9	88	75.2
Education	Primary	29	5.8	2	1.7
	Vocational	142	28.4	12	10.3
	Secondary	176	34.9	50	42.7
	Higher	155	30.9	53	45.3
Employment	Working	264	52.6	68	58.1
	Out-of-work	238	47.4	49	41.9
Partner status	In relationships	359	78.9	69	59
	Single	143	21.1	48	41
Having children	Yes	396	79.8	76	65
	No	106	20.2	41	35
Faith	Believer	451	89.8	102	87.2
	Non-believer	51	10.2	15	12.8

**Table 2:** Basic descriptive statistics of psychological variables in the studied groups.

	Clinical Group					Control Group				
Variable	*M*	*SD*	*Me*	*Min*	*Max*	*M*	*SD*	*Me*	*Min*	*Max*
**CISS**										
Task-Oriented	51.39	9.10	51.00	23	76	55.32	8.84	55.00	35	75
Avoidance-Oriented	44.82	7.89	45.00	17	70	46.74	8.60	47.00	20	76
Emotion-Oriented	44.50	9.40	45.00	20	74	43.54	10.35	43.00	20	75
Distraction	20.91	5.04	21.00	8	38	21.57	5.12	21.00	8	38
Social Diversion	15.61	3.66	16.00	6	25	16.40	3.70	16.00	8	25
**MHLC**										
Internal	23.42	5.11	23.00	7	36	25.06	4.78	25.00	11	36
Powerful Others	23.64	4.97	23.00	11	36	22.24	4.87	22.00	6	36
Chance	22.80	5.74	23.00	7	36	20.36	5.92	20.00	8	31
**MAAS**	58.24	12.92	58.00	16	90	61.70	13.59	64.00	30	85

**Table 3:** Differences in psychological variables between the clinical group and the control group.

Variable	*R*		U	*p*
	Control Group (*N* = 117)	Clinical Group (*N* = 502)		
CISS Task-Oriented	369.37	296.16	22.421	<0.001
CISS Avoidance-Oriented	342.87	302.34	25.521	<0.05
CISS Emotion-Oriented	292.96	313.97	27.373	0.252
CISS Distraction	324.10	306.71	27.717	0.342
CISS Social Diversion	336.35	303.86	26.283	0.075
MHLC Internal	357.66	298.89	23.791	<0.001
MHLC Powerful Others	281.96	316.53	26.086	<0.05
MHLC Chance	254.19	323.01	22.837	<0.001
MAAS	350.71	300.51	24.603	<0.01

R-ranks; U-Mann-Whitney U Test; p-significance level.

## Data Availability

All data are presented in the manuscript.
